# Receptor-associated prorenin system contributes to development of inflammation and angiogenesis in proliferative diabetic retinopathy

**DOI:** 10.1186/s41232-016-0027-0

**Published:** 2016-09-07

**Authors:** Atsuhiro Kanda, Susumu Ishida

**Affiliations:** Laboratory of Ocular Cell Biology and Visual Science, Department of Ophthalmology, Hokkaido University Graduate School of Medicine, N-15, W-7, Kita-ku, Sapporo, Hokkaido 060-8638 Japan

**Keywords:** Receptor-associated prorenin system, Renin-angiotensin system, Angiotensin II type 1 receptor, (Pro)renin receptor

## Abstract

The renin-angiotensin system (RAS) plays a potential role in the development of end-organ damage, and tissue RAS activation has been suggested as a risk factor of several diseases including diabetes. So far, using animal disease models, we have shown molecular mechanisms, in which tissue RAS stimulates retinal angiogenesis, and the critical roles of (pro)renin receptor [(P)RR] in retinal RAS activation and its concurrent intracellular signal transduction, referred to as the receptor-associated prorenin system (RAPS). Moreover, we recently reported that the protein levels of prorenin and soluble (P)RR increased in the vitreous fluids obtained from patients with proliferative diabetic retinopathy (PDR), suggesting the association of (P)RR with vascular endothelial growth factor (VEGF)-driven angiogenic activity in human PDR, and also showed a close relationship between the vitreous renin activity and VEGF-induced pathogenesis of diabetic retinopathy. Our data using animal disease models and human clinical samples suggest that both vitreous RAS and retinal RAPS play critical roles in the molecular pathogenesis of diabetic retinopathy.

## Background

Diabetic retinopathy (DR) is one of the severe complications of diabetes and leading cause of severe vision loss and blindness when it progresses to the stage of proliferative DR (PDR) characterized by fibrovascular proliferation. Fibrovascular tissue develops by the extension of retinal angiogenesis into the vitreous cavity, and formation of the fibrovascular tissue results in severe complications, such as tractional retinal detachment and vitreous hemorrhage. Several growth factors and cytokines are involved in the molecular pathogenesis of diabetic retinopathy; however, vascular endothelial growth factor (VEGF) has been considered as the major angiogenic and proinflammatory factor in PDR [1–3]. VEGF plays important roles in normal physiology such as in embryogenesis, endometrial maturation, and wound healing. However, it also causes profound pathogenesis complicating diabetes and cancer. Tumor growth requires new vessel formation, which is driven predominantly by VEGF, the most potent angiogenic factor and the principal target for anti-angiogenic therapy [4]. We previously revealed a significant contribution of VEGF165 isoform to angiogenic activity in PDR, showing that fibrovascular tissues co-expressing VEGF receptor (VEGFR)-2 and neuropilin (NRP) 1, the specific receptor for VEGF165, were highly vascularized [5–7]. VEGF165 were shown to increase the expression of adhesion molecules and subsequently stimulates leukocyte infiltration leading to the development of retinal angiogenesis [5–7].

The renin-angiotensin system (RAS), a known important controller of systemic blood pressure (circulatory RAS), plays distinct roles in inflammation and pathological vascular conditions in organs including the brain, eye, heart, liver, and kidney (tissue RAS) [8]. Tissue RAS acts in a paracrine fashion and regulates various biological and pathological events such as cell signaling, apoptosis, proliferation, angiogenesis, immune responses, and extracellular matrix formation [9–11]. In this review, we focus on the relationship between diabetic retinopathy and tissue RAS and suggest a novel concept for the molecular pathogenesis of tissue RAS in the vitreous, referred to as “vitreous RAS.”

## Vitreous renin-angiotensin system and retinal receptor-associated prorenin system in diabetic retinopathy

Several types of organ damage are known to result from activation of tissue RAS. As concerns its relationship with the eye, pharmacological blockade of angiotensin-converting enzyme (ACE) or angiotensin II type 1 receptor (AT1R) resulted in beneficial effects on the incidence and progression of DR in several clinical trials including the EUCLID study, DIRECT-Prevent 1, DIRECT-Protect 1, DIRECT-Protect 2, and the RAS study [12–15]. We unraveled the molecular mechanisms in which tissue RAS causes retinal inflammation and angiogenesis in the murine model of endotoxin-induced uveitis, strepotozotocin-induced diabetes, and laser-induced choroidal neovascularization [16–18] and the critical role of (pro)renin receptor [(P)RR] in retinal RAS activation [19–22]. Tissue RAS is initiated by prorenin binding with (P)RR to acquire renin activity, which also causes RAS-independent signal transduction in cells bearing (P)RR. Prorenin binding to (P)RR causes renin activity through the conformational change of prorenin (non-proteolytic activation of prorenin causing tissue RAS) instead of the conventional proteolysis of the prorenin prosegment by processing enzymes (proteolytic activation of prorenin causing circulatory RAS). In addition to tissue RAS activation, prorenin binding to (P)RR activates RAS-independent signal transduction via mitogen-activated protein kinases including extracellular signal-regulated kinase (ERK) 1/2 pathway, which has been shown to contribute to organ damage. (P)RR can bind to both prorenin and renin, but the binding affinity of prorenin is much higher than that of renin [23]. The (P)RR-mediated dual activation of tissue RAS and RAS-independent signaling pathways, referred to as the receptor-associated prorenin system (RAPS), was shown to be involved in the molecular pathogenesis of ocular disorders including retinal inflammation and choroidal neovascularization [20, 21, 24], both of which are due to the upregulated expression of VEGF in the downstream of retinal and choroidal RAPS, respectively.

**Fig. 1:**
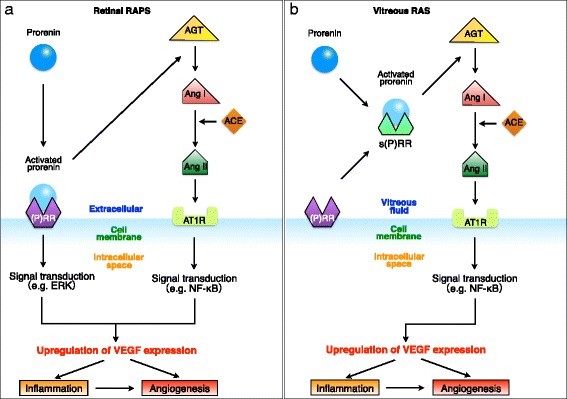
A schema showing the significant involvement of retinal RAPS (**a**) and vitreous RAS (**b**) with the VEGF-driven pathogenesis of diabetic retinopathy. Vitreous RAS is caused by s(P)RR, whereas retinal RAPS depends on membrane-type (P)RR (modified from Kanda et al. [27]). *ACE* angiotensin-converting enzyme, *AGT* angiotensinogen, *Ang I* angiotensin I, *Ang II* angiotensin II, *AT1R* angiotensin II type 1 receptor, *ERK* extracellular signal-regulated kinase, *NF-kB* nuclear factor-kB, *(P)RR* (pro)renin receptor

Remarkably, (P)RR was reported to undergo cleavage by proteases to generate a soluble form of (P)RR [s(P)RR], whereas it still has a capability for non-proteolytic activation of prorenin, causing the conversion of angiotensinogen (AGT) to angiotensin I (Ang I) in vitro [25]. We have shown that s(P)RR, prorenin, activated prorenin, and VEGF protein levels together with renin activity levels in vitreous fluids were significantly higher in PDR eyes compared to non-diabetic controls [26, 27]. Increased protein levels of s(P)RR in PDR eyes, released from neovascular endothelial cells in fibrovascular tissues, were significantly correlated with vitreous prorenin, activated prorenin, and VEGF protein levels and the vascular density of fibrovascular tissues [26]. Interestingly, renin activity levels also significantly correlated with the vitreous protein levels of s(P)RR, prorenin, activated prorenin, and VEGF [27]. These data indicate that the vitreous renin activity stems from s(P)RR-mediated non-proteolytic activation of prorenin, suggesting the significant role of (P)RR in the pathogenesis of PDR. Indeed, (P)RR and RAS components were expressed in diabetic fibrovascular tissues, human retinal cell lines, and normal ocular tissues [26, 28–30], and the vitreous levels of prorenin and angiotensin II (Ang II) were shown to be elevated in PDR eyes [31–34]. Furthermore, a close link between the vitreous renin activity and VEGF protein levels validates our concept of vitreous RAS that contributes to the angiogenic activity of DR. Consequently, in concert with vitreous RAS due to s(P)RR (Fig. 1a) [27], retinal RAPS due to membrane-type (i.e., full-length) (P)RR [26] (Fig. 1b) is thought to regulate VEGF expression in DR. Moreover, we have recently shown that RAPS is involved in the molecular pathogenesis of organ damage, such as inflammation, angiogenesis, and fibrosis, including conjunctival lymphoma [28] and other ocular disorders (under review).

Although we have shown the significant role of (P)RR signaling via ERK [21, 26] as well as AT1R signaling via nuclear factor (NF)-kB [16] in the upregulation of VEGF expression, it is difficult to determine the ratio of involvement with the angiogenic activity in human PDR. Cleavage enzymes for processing full-length (P)RR to s(P)RR include the proprotein convertase furin [35] and ADAM (a disintegrin and metalloproteinase) 19 [36], both of which proved to be present in endothelial cells in the fibrovascular tissue in PDR [26]. Gene expression and enzymatic activity of these proteases in the neovascular endothelial cells are likely to define the contribution ratio between vitreous RAS and retinal RAPS. Investigation into the biochemical regulation of furin and ADAM19 is required in the future to further elucidate (P)RR-related molecular pathogenesis of diabetic retinopathy.

The significance of the pathogenic system vitreous RAS may be attributed in part to a possibility of revising the current surgical indication and concept of vitrectomy for DR. In clinical setting, retinal surgeons remove the vitreous from PDR eyes because of (1) vitreous hemorrhage from newly formed vessels disturbing the visual axis and (2) tractional retinal detachment in which the retina is elevated by the vitreous that functions as the scaffold of the fibrovascular proliferative tissue originating from retinal vessels. These two major classic indications to the advanced stage have long been applied in terms of a mechanical or physical cue. In contrast, our data on vitreous renin activity indicate the possibility of the vitreous per se as the amplifier of the molecular pathogenesis of PDR. Retinal surgeons frequently encounter surgical cases where diabetic macular edema, a consequence of VEGF-induced vascular hyperpermeabiliy, is diminished soon after vitrectomy. This is explained at least in part by the pathological concept of vitreous RAS, the driving force of the downstream AT1R/nuclear factor-kB (NF-kB)/VEGF axis responsible for the pathogenesis of diabetic retinopathy (Fig. 1). It is reasonable, therefore, to think that the vitreous is not just the reservoir of detrimental cytokines but the factory of pathogenic RAS components. In this sense, vitrectomy procedure harbors a biochemical implication, which may expand the current surgical strategy to earlier intervention for broader indications to reduce the vitreous RAS-derived capability of producing VEGF and other several cytokines.

## Conclusions

Our findings may not only lead to a new understanding of the molecular pathogenesis that implies a close link among the vitreous RAS, retinal RAPS, and VEGF-induced pathogenesis of diabetic retinopathy but also activate the clinical research in the surgical as well as medical point of view, thus contributing to further improvement of visual prognosis in patients with DR.

## Abbreviations

(P)RR: (Pro)renin receptor
ACE: Angiotensin-converting enzyme
AGT: Angiotensinogen
Ang: Angiotensin
AT1R: Angiotensin II type 1 receptor
DR: Diabetic retinopathy
ERK: Extracellular signal-regulated kinase
NF-kB: Nuclear factor-kB
PDR: Proliferative diabetic retinopathy
RAPS: Receptor-associated prorenin system
RAS: Renin-angiotensin system
VEGF: Vascular endothelial growth factor
